# Supplementary material for: 2D-Hexagonal Boron Nitride Screen-Printed Bulk-Modified Electrochemical Platforms Explored towards Oxygen Reduction Reactions
Source: Sensors (Basel). 2022 Apr 26;22(9):3330. doi: 10.3390/s22093330 (PMC9105127; doi:10.3390/s22093330)

Electronic Supporting Information (ESI) for the following publication:

2D-Hexagonal Boron Nitride Screen-Printed Bulk Modified Electrochemical Platforms Explored Towards the Oxygen Reduction Reaction

Aamar F. Khan[1,3], Alejandro Garcia-Miranda Ferrari[1,3], Jack P. Hughes[1,3], Graham C. Smith[2], Craig E. Banks[1,3] and Samuel J. Rowley-Neale[1,3]*

[1]: *Faculty of Science and Engineering, Manchester Metropolitan University, Chester Street, Manchester M1 5GD, UK.*

[2]: *Faculty of Science and Engineering, Department of Natural Sciences, University of Chester, Thornton Science Park, Pool Lane, Ince, Chester CH2 4NU, UK.*

[3]: *Manchester Fuel Cell Innovation Centre, Manchester Metropolitan University, Chester Street, Manchester M1 5GD, UK.*

*To whom correspondence should be addressed.

S.J.R.-N. / Email: s.rowley-neale@mmu.ac.uk

C.E.B. / Email: c.banks@mmu.ac.uk; Tel: ++(0)1612471196; Fax: ++(0)1612476831;

Website: www.craigbanksresearch.com

Figure S1. (A) A typical Raman spectra obtained for the commercially procured 2D-hBN immobilised upon a supporting silicon wafer. (B) Typical TEM images of the 2D-hBN deposited onto a holey carbon film supported upon a Cu TEM grid. Scale bars are 200 nm. Images obtained using a 200 kV primary beam under bright-field conditions. (C) XRD spectra of the 2D-hBN, exhibiting characteristic peaks at 26.7°, 41.56° and 44.38°.

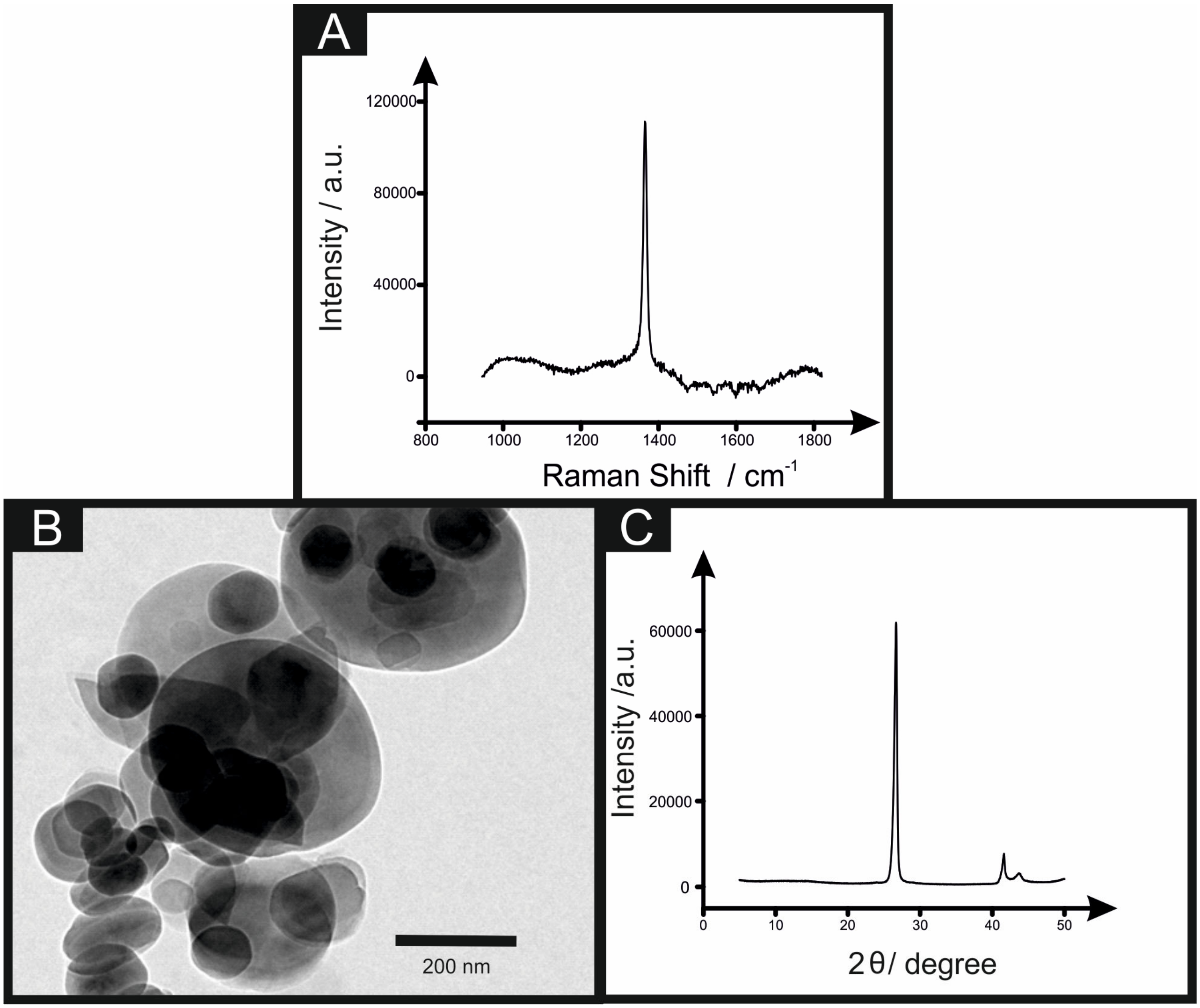

Figure S2. (A) Scanning Electron Microscopy (SEM) image of the pristine 2D-hBN powder on a Si wafer utilised to fabricate the 2D-hBN/SPEs studied herein. (B) Energy Dispersive X-Ray Spectroscopy (EDS) composition analysis of a 2D-hBN flake. This is a representative image obtained from 'batch characterisation' of the samples that have been previously utilised in Ref [48].

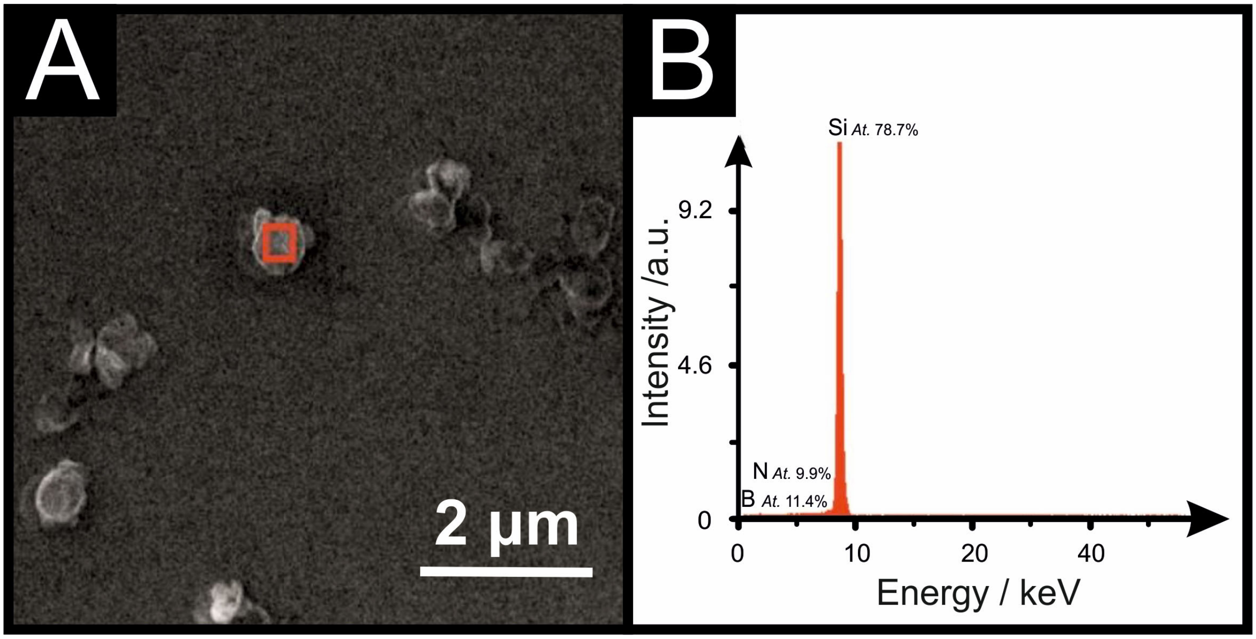

Figure S3. (A) X-ray photoelectron spectroscopy (XPS) analysis of the pristine 2D-hBN powder on a Si wafer utilised to fabricate the 2D-hBN/SPEs studied herein. High resolution analysis of the B 1s and N 1s components (B and C respectively).

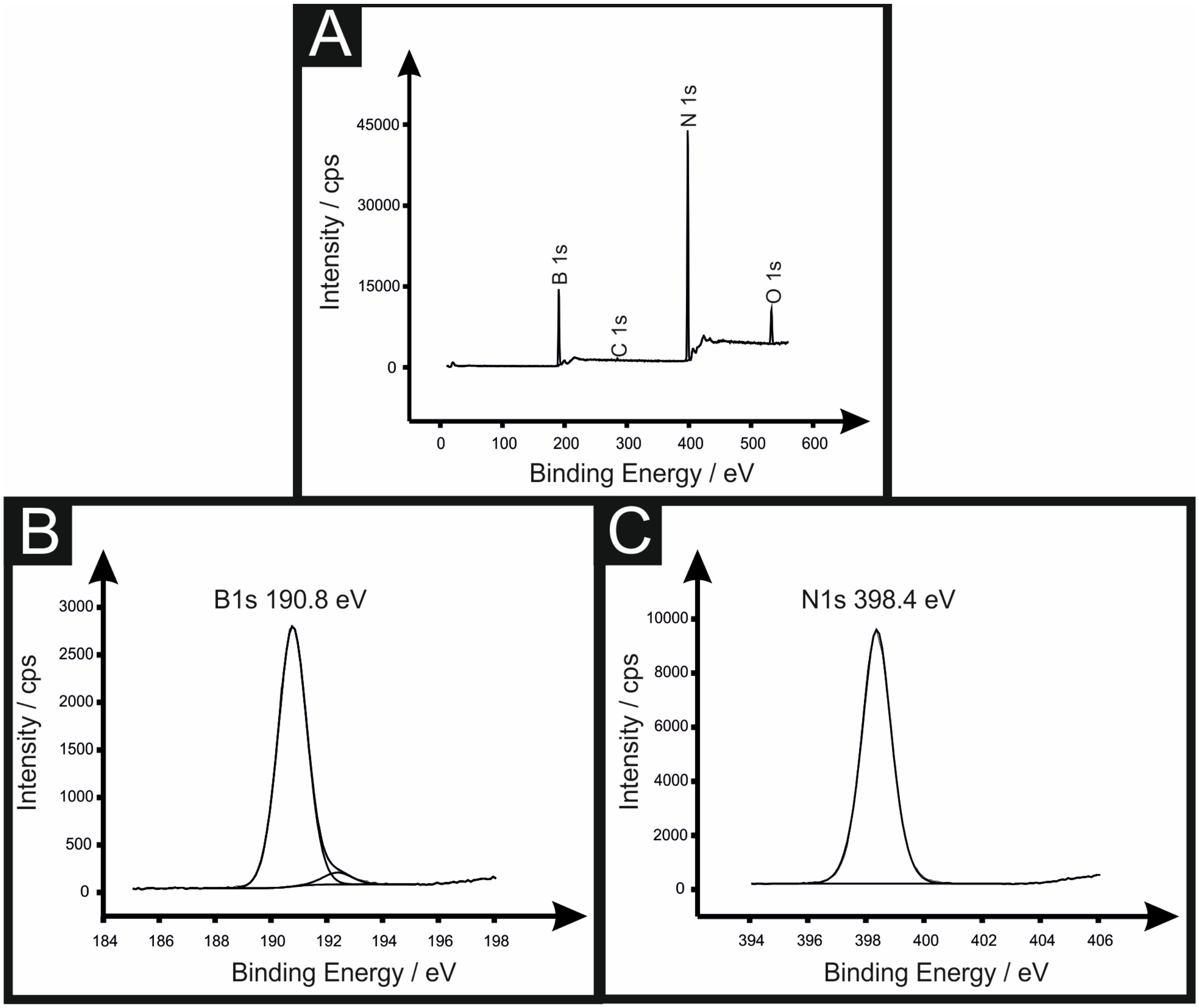

Figure S4. Typical linear sweep voltammograms (LSVs) recorded in an oxygen saturated 0.1 M H_2SO_4 solution using a macro polycrystalline Pt electrode**.** Scan rate: 100 mV s^{-1} (*vs*. SCE).

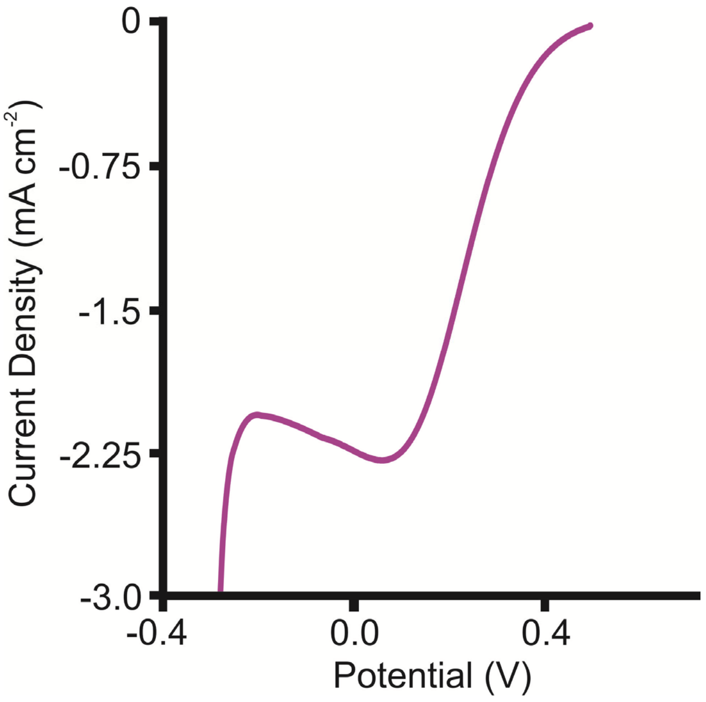

Supplement: Supplementary file 1 [file sensors-22-03330-s001.zip › sensors-1542642-supplementary.pdf]
